# Orthopœdic Surgery; Wm. Wood & Co.; Keating’s Cyclopedia, Vol. III

**Published:** 1890-07

**Authors:** 


### Book Notices.

A TREATISE ON ORTHOPEDIC SURGERY, by Edward H. Bradford, M. D., Surgeon to Children's Hospital, Boston City Hospital and Samaritan Hospital, instructor in Clinical Surgery, Harvard Medical School; and Robert W. Lovett, M. D., Surgeon to the Samaritan Hospital, Assistant Out-patient Surgeon to the Children's Hospital, Out-patient Surgeon to the Army Hospital, formerly Assistant Surgeon to the New York Orthopedic Dispensary and Hospital. 783 pages, illustrared with 789 wood engravings. New York: Wm. Wood & Co. 1890.

This is a splendid book; worth its weight in gold to every surgeon.

It is only within the last two decades that orthopedic surgery has become recognized as a distinct branch of practice. In that time the progress made has been truly wonderful, and many deformities of the lower extremities heretofore thought to be beyond relief, have been corrected by operation. This has become possible since the days of antiseptic surgery. The cure of flat foot is the latest triumph; the arch is altered and remodeled as that of a bridge would be, and insteps and graceful feet are now made to order.

In writing this excellent work, the authors have not confined themselves strictly to orthopedic surgery as generally understood to mean the correction of existing deformities of the lower extremities, but have entered into a description and discussion of affections of the joints as factors in the production of deformity, holding that prevention, as well as cure, is the mission of the surgeon; and having opened up on this branch, it was hard to draw a line; hence Pott's disease, which is supposed to have no relation to orthopedics, is treated in the opening chapter, and extensively illustrated. Deformities resulting from traumatism are not considered in this work, the ground being fully covered in works on general surgery.

The title of the work does not convey an adequate idea of its scope; it is a misnomer. The first one hundred pages are devoted to Pott's disease; the next one hundred to lateral curvature and other affections of the spine; a large part to paralysis, and a part to torticollis; while only the latter one-third of the volume is devoted to orthopedic surgery proper. It is a much bigger book than "orthopedic surgery"; it is an exhaustive treatise on the "cause, prevention and treatment of all kinds of congenital or acquired deformities of the human osseous or muscular system." Moreover, it is published under the general head of "specialties in the practice of medicine." We cannot exactly understand where the relation to the practice of medicine comes in. But, as before said, it is a very valuable book. The publishers, Messrs. Wood & Co., have, with their usual painstaking care, done their share in getting it out. It is a handsome volume in muslin. Price, $6.

---

CYCLOPÆDIA of the Diseases of Children, Medical and Surgical. Keating, Vol. III. Diseases of the Digestive System; Of the Genito-Urinary Organs and of the Blood. Surgery and Diseases of the Osseous System, and of the Joints: J. B. Lippincott & Co., Philadelphia, 1890.

The articles written especially for the work by American, British and Canadian authors. Edited by Jno. |O. Keating, M. D.; illustrated.

This is the third volume in the series—the first of which created an unusual interest in medical circles, and its get up, and the quality of its contents fully sustain the fine impression made by Vols. I and II. The articles which go to make up the first part of this volume, and embraced under the head of Diseases of the Digestive System, are numerous and ably written. They cover the ground in a most exhaustive manner, and furnish the practitioner with new views and new treatment; something out of the ordinary, stereotyped style. Every phase of the subject is ably written up by men who have had unusual facilities for observation, and who are abundantly able to profit by that observation, and the soundness of whose deductions from clinical facts is beyond question.

Beginning with Functional Disorders of the Stomach, by Pepper, the reader is carried by easy stages and regular gradation successively through—Diseases of the Stomach (Hobart Amerry Hart), the Diarrhœal Diseases, acute and chronic (L. Emmett Holt), Membranous Enteritis (Wm. A. Edward), Intestinal Bacteria of Children (Wm. D. Booker), Acute Chronic Constipation (Chas. W. Earle), Tabes Mesinterica (A. Jacobi), Parasites of the Intestinal Canal (W. T. Councilman), Intestinal Obstruction in Children (W. W. Keen), Petitonitis (Henry Ashby), Perityphlitis, Paratyphlitis and Perityphlitic Abscess, (Christian Fenger), Congenital Abnormalities of the Intestines, Diseases of the Rectum and Anus (Henry R. Wharton), Colotomy (Jno. H. Packard), etc. A chapter is devoted to the pancreas, and three articles on the liver.

This is equal to a full course of lectures on the subject, and were there nothing else in the book, would be worth the price of the set.

Part II is devoted to Diseases of the Genito-Urinary Organs and the Blood. Part III to Surgery, embracing Minor Surgery and Emergencies in Children, by Dr. C. W. Dulles. Plastic Surgery by T. G. Morton; Wounds by Jas. McCann; and Anæsthetics and Anæsthesia by O. H. Allis. Part IV, to the Diseases of the Osseous System and of the Joints.

To the latter subject fifteen well written articles are devoted, by such men as Gerster, MacEwen, (Glasgow), Kinlock, (Charleston), N. P. Gibney, A. J. Steele, et al.

The whole is profusely illustrated. The work in its entirety, is a masterpiece; it is the embodiment of scientifiic teaching, and brings the reader up with the latest accepted doctrines on the subjects treated cf. What a privilege to live and practice in such an age, with such facilities for acquiring knowledge! The vast body of the profession jog along in an easy way, while such men as the writers of this book are delving into the mines of knowledge, waiting, knowing, that soon the fruits of their labor will be sifted, formulated and systematically arranged and presented to them in a neat and attractive form, and at a triflng cost; and all they have to do is to—enjoy it and profit by, and give the afflicted the benefit of said "delving." We of the profession stand under the tree with hands outstretched to catch the cherries;—others have climbed the tree and do the shaking.

Messrs. Lippincott & Co., seem to have taken a special pride in getting up the work in a style commensurate with the great value of its contents. It is a book that any physician should be proud to possess; it is intensely interesting. We regard it as one of the most valuable and important additions to literature within the century.

---

TALES OF A COUNTRY DOCTOR, by Dr. Willis P. King, Kansas City.

Readers, here is a treat for you. It is full of pathos and humor, and of a wholesome kind of satire. It is full of the milk of human kindness, and only lacks a little of being a first-class sermon on ethics. Send for it. Some of the Doctor's experiences will find echo in many breasts.

